# An Investigation of the Wild Rat Crown Incisor as an Indicator of Lead (Pb) Exposure Using Inductively Couple Plasma Mass Spectrometry (ICP-MS) and Laser Ablation ICP-MS

**DOI:** 10.3390/ijerph18020767

**Published:** 2021-01-18

**Authors:** Andrew Kataba, Shouta M. M. Nakayama, Hokuto Nakata, Haruya Toyomaki, Yared B. Yohannes, John Yabe, Kaampwe Muzandu, Golden Zyambo, Ayano Kubota, Takehisa Matsukawa, Kazuhito Yokoyama, Yoshinori Ikenaka, Mayumi Ishizuka

**Affiliations:** 1 Laboratory of Toxicology, Department of Environmental Veterinary Sciences, Faculty of Veterinary Medicine, Hokkaido University, Kita 18 Nishi 9, Kita-ku, Sapporo 060-0818, Japan; andrewkataba@gmail.com (A.K.); hokuto.nakata@vetmed.hokudai.ac.jp (H.N.); yahahuhru@gmail.com (H.T.); ybyared@gmail.com (Y.B.Y.); y_ikenaka@vetmed.hokudai.ac.jp (Y.I.); 2 School of Veterinary Medicine, The University of Zambia, P.O. Box 32379, Lusaka 10101, Zambia; mjyabe@yahoo.co.uk (J.Y.); kmuzandu@yahoo.com (K.M.); goldzgambo@gmail.com (G.Z.); 3 Department of Chemistry, College of Natural and Computational Science, University of Gondar, P.O. Box 196, Gondar 6200, Ethiopia; 4 Department of Epidemiology and Environmental Health, Faculty of Medicine, Juntendo University, 2-1-1 Hongo, Bunkyo-ku, Tokyo 113-8421, Japan; ay-kubota@juntendo.ac.jp (A.K.); tmatsuka@juntendo.ac.jp (T.M.); kyokoya@juntendo.ac.jp (K.Y.); 5 Water Research Group, School of Environmental Sciences and Development, North-West University, Private Bag X6001, Potchefstroom 2531, South Africa

**Keywords:** lead, incisor, biomarker, sentinel, wild rodent

## Abstract

Lead (Pb) is a metal toxicant of great public health concern. The present study investigated the applicability of the rat incisor in Pb exposure screening. The levels of lead in teeth (Pb-T) in the crown and root of incisors in laboratory Pb-exposed Sprague Dawley rats were quantified using inductively coupled plasma mass spectrometry (ICP-MS). The crown accumulated much Pb-T than the root of the Sprague Dawley rat incisor. The levels of lead in blood (Pb-B) were positively correlated with the Pb-T in the crown and root incisors of the Sprague Dawley rats. As an application of the Pb-T crown results in experimental rats, we subsequently analyzed the Pb-T in the crown incisors of Pb-exposed wild rats (*Rattus rattus*) sampled from residential sites within varying distances from an abandoned lead–zinc mine. The Pb-T accumulation in the crown of incisors of *R. rattus* rats decreased with increased distance away from the Pb–Zn mine. Furthermore, the Pb-T was strongly correlated (*r* = 0.85) with the Pb levels in the blood. Laser ablation ICP-MS Pb-T mappings revealed a homogenous distribution of Pb in the incisor with an increased intensity of Pb-T localized in the tip of the incisor crown bearing an enamel surface in both Sprague Dawley and *R. rattus* rats. These findings suggest that Pb-T in the crown incisor may be reflective of the rat’s environmental habitat, thus a possible indicator of Pb exposure.

## 1. Introduction

Lead (Pb) is a toxic metal known to cause a number of physiological and biochemical dysfunctions in animals and humans [1]. Although Pb poisoning has considerably receded in developed countries [2], chronic exposure to low levels of Pb remains a perennial phenomenon in some developing countries. A case in point is Kabwe town in Zambia, with a lead–zinc mine history legacy characterized by an alarming Pb poisoning in adults and children living near the closed lead–zinc mine [3,4,5]. Moreover, acute fatal cases of Pb poisoning in over 400 children in Nigeria [6] and 18 children in Dakar, Senegal [7] linked to anthropogenic activities have been reported. Even at low non-fatal levels, Pb exposure causes cognitive impairments in children exposed in early life stages [2]. In adults, low cumulative Pb exposure has been linked to renal, cardiovascular, and reproductive-system-related disorders [8,9]. Bellinger et al. [8] further revealed that early stage Pb exposure in children led to disadvantaged adult outcomes such as poor academic performance and low income. Thus, biomarkers of Pb exposure that are reliable and easy to obtain from humans and sentinel animals that share habitats with humans are required [10].

Traditionally, Pb in blood (Pb-B) has been widely used as a biomarker for Pb exposure in humans [11,12]. However, Pb-B poses a short mean biological life of only around 30–40 days and may only reflect primarily both ongoing steady-state exposures and relatively recent exposures [10]. Moreover, changing the conditions of exposure causes a Pb-B variation and typically the blood Pb level reverts to normal once the exposure ceases [13]. In contrast, the concentration of Pb in teeth (Pb-T) is a cumulative function of earlier exposure which allows for the identification of historic undetected cases of Pb exposure even after the other indices have returned to normal [14]. Although the Pb-T in the whole tooth has been considered as a biomarker of Pb exposure in children [15] and rodents [16], the distribution Pb within is not even. The uneven distribution of Pb in teeth may be due to different dental structures and the level of calcification of the dental parts [12].

The use of laser ablation inductively coupled plasma mass spectrometry (LA-ICP-MS) as a technique has been applied to estimate the distribution patterns of metals in the dentine and enamel parts of the teeth [17,18,19]. However, there are limited reports on the distribution or mapping of Pb in rodent incisor teeth of wild or laboratory rats exposed to Pb. Therefore, further investigations using techniques for advanced mapping of distribution Pb in teeth such as LA-ICP-MS are needed.

Wild rodents have been used as sentinel animals and biomonitors of environmental-related pollution assessments of pesticides [20], asbestos [21], and heavy metal pollution [22]. In the present study, the rodent incisor tooth was investigated in laboratory and wild rodents as an indicator of Pb exposure. We hypothesized that Pb accumulates differently within the incisor teeth and that the part with much accumulation may be sampled as a biomarker of Pb exposure. To the best of our knowledge, the quantification of Pb-T in the root and crown subdivisions of incisor teeth in Pb-exposed laboratory rats and the use of the incisor crown of wild rats as an indicator of Pb exposure has never been reported. Lead distribution mappings were performed using LA-ICP-MS in both laboratory and wild rats exposed to Pb to augment the Pb-T quantification done using ICP-MS and ascertain the distribution of Pb in rodent incisor. The purpose of this study was to evaluate the accumulation pattern of lead in the crown and root incisor teeth of laboratory rats following lead exposure. Based on the laboratory rat results that showed high levels of Pb in the crown incisor, the study explored the use of the wild rat crown incisor as an indicator of lead exposure in a field situation. Furthermore, the distribution pattern of lead in rodent incisor using laser ablation inductively coupled plasma mass spectrometry was evaluated.

## 2. Materials and Methods

### 2.1. Laboratory Animals and Exposure

Animal experiments were performed at the Faculty of Veterinary Medicine, Hokkaido University under the supervision and with the endorsement of the Institutional Animal Care and Use Committee of Hokkaido University, Sapporo, Japan (approval number: 16-0017). Male Sprague Dawley rats (*n* = 18) aged seven weeks were purchased from Sankyo Labo Service Corporation, Inc. (Tokyo, Japan). The rats were kept in six lead-free polypropylene cages in community housing of three rats (*n* = 3 per cage). The animals were acclimated to the animal facility for one week prior to Pb exposure with access to lead-free food (rodent chow, Labo MR Stock, Nosan Corporation, Yokohama, Japan) and distilled water *ad libitum*. There were no significant body weight differences among all the groups. Two cages with six (*n* = 6) rats were randomly assigned to the three exposure levels (control, low, and high dosage exposure). Two different concentrations of Pb acetate, 100 mg/L and 1000 mg/L Pb (Wako Pure Chemical Industries, Osaka, Japan), were given through drinking water for eight weeks to the low and high dosage groups, respectively. The control group received only distilled water for the same period. The choice of Pb levels of exposure for the current study was based on the previous study in mice that had a dose-dependent Pb tissue accumulation at 100 mg/L and 1000 mg/L lead acetate concentrations [23]. At the end of the exposure period, rats were euthanized under carbon dioxide with sevoflurane. Blood and incisors teeth (upper and lower) were collected following extraction. For uniformity, we assigned the upper and lower incisors on the left side of the jaws for quantitative Pb analysis using inductively coupled plasma mass spectrometry (ICP-MS), and those on the right side were assigned for qualitative Pb mapping using LA-ICP-MS for each individual rat across all the groups. The blood and teeth samples were collected in lead-free polypropylene tubes and were kept at −80 °C and −20 °C prior to analysis, respectively.

### 2.2. Wild Rat Sampling and Species Identification

**Figure 1:**
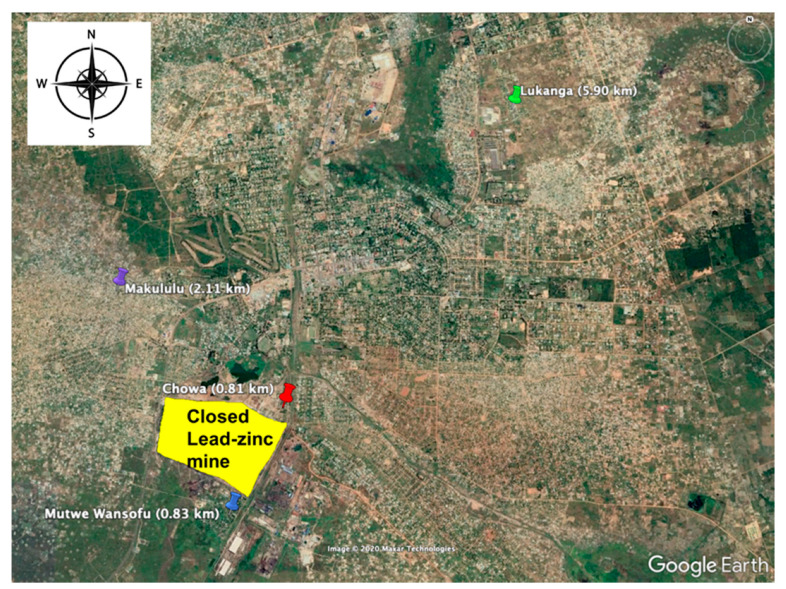
Sampling sites for wild rats. Mutwe Wansofu (MW; *n* = 6), Chowa (CH; *n* = 6), Makululu (MK; *n* = 6), and Lukanga (LK; *n* = 6). The closed Pb–Zn mine is shown with a “yellow” shape.

The sampling was done with permission and strict adherence to the guidelines from the Zambian Ministry of Fisheries and Livestock, as well as the Faculty of Veterinary Medicine, Hokkaido University, Sapporo, Japan (approval number: Vet-17010). Wild rats were collected from Kabwe, Zambia, a town known for extensive Pb environmental contamination [4,5,24,25] between June and July 2017. Kabwe is situated at approximately 142°70′ S and 28°260′ E. Four residential townships in Kabwe, namely, Lukanga (LK), Makululu (MK), Chowa (CH), and Mutwe Wansofu (MW) were used as sampling sites for wild rats based on their relative distances to the closed lead–zinc (Pb–Zn) mine. Six (*n* = 6) wild rats were captured using live traps as previously described by our research group [25] from each of the four sites within varying distance from the closed Pb–Zn mine. The furthest distance away from the closed mine in Lukanga (LK; 5.90 km) was used as the control site. The other sites closer to the closed mine were taken as exposed sites, namely, Makululu (MK; 2.11 km), Chowa (CH; 0.86 km), and Mutwe Wansofu (MW; 0.81 km), respectively. The sampling sites were located and identified (Figure 1) using a global positioning system (GPS). The rats were euthanized with sevoflurane and blood and incisors teeth (lower) were collected following extraction. The samples were then immediately stored at –20 °C before being transported to the Faculty of Veterinary Medicine, Hokkaido University Sapporo, Japan under the cold chain systems. The transportation of samples was done in accordance with the international sample passage certificate from the Ministry of Fisheries and Livestock, Zambia (No. 11614). At Hokkaido University, the blood and teeth samples were stored at −80 °C and −20 °C in a deep freezer, respectively, until analysis. The species of the wild rats were identified using the genomic DNA sequencing method previously described by Robins et al. [26], and only rats that were of *Rattus rattus* species (Supplementary Materials, Table S1) were used in the current study. After the exclusion of the non-*R. rattus* rat species from the six (*n* = 6) rats trapped from each site, the final sample sizes were *n* = 2 (LK), *n* = 5 (CH), *n* = 5 (MK), and *n* = 3 (MW), respectively. A detailed description of the methods of species identification can be found in Appendix A.

### 2.3. Digestion and Quantitative Analysis of Lead in Blood and Teeth Subdivisions

**Figure 2:**
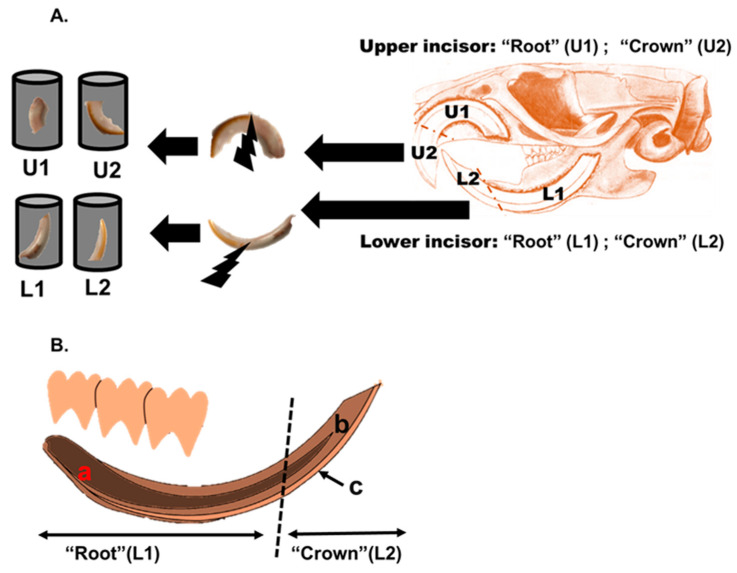
Divisions of the incisor teeth and schematic illustration of the longitudinal section of the incisor tooth. (**A**) Lower and upper incisor tooth divisions based on the yellow-orange enamel for microwave acid digestion. For upper incisors, the root (U1) part is anchored in the maxillary bone, and the crown (U2) part of the tooth is seen externally. Similarly, for lower incisors, the root (L1) part is anchored in the mandibular bone, and the crown (L2) part of the tooth is seen externally. (**B**) Illustrated schematic longitudinal section modified from Park et al. [31] of lower incisor tooth as was sectioned for direct LA-ICP-MS analysis. Furthermore, the three major parts of the incisor tooth are seen—(a) dental pulp, (b) dentine, and (c) enamel running only in the front part of the tooth. The dotted line is the imaginary division line used based on the obvious discoloration of enamel on the crown part of the tooth.

The Pb concentration in the blood and incisors teeth collected from the laboratory exposed Sprague Dawley rats and the *R. rattus* rats from Kabwe, Zambia were quantified. In the current study, blood and teeth were digested using the microwave acid method previously described by Nakata et al. [27,28] with minor modifications. To minimize surface contamination and remove excess blood, the teeth were firstly cleaned using an ultrasonic bath method as described by Ishii et al. [29] with some modifications. Briefly, the teeth samples were placed in an ultrasonic bath of L-cysteine (Cica reagent, 100 mg/L; Kanto Chemical, Tokyo, Japan) for 5 min followed by rinsing in an ultrasonic bath of distilled water for 10 min prior to drying. The samples were then dried in an oven at 54 °C for 48 h. Each tooth was divided into two subdivisions using the yellow-orange pigmentation of enamel on the crown part of the labial surface of the tooth [30] as a distinguishing reference mark as illustrated in Figure 2. Each lower (L) incisor was divided into two subdivisions, namely, L1 (root) and L2 (crown). The L1 is a part of the tooth that is embedded in the jawbone and L2 for the part tooth visible in the oral cavity. Similarly, the upper (U) incisor was divided into two subdivisions, namely, U1 (root) for the upper part of the tooth embedded in the jawbone and U2 (crown) for the part tooth visible in the oral cavity in live rodents as shown in Figure 2. In the case of the blood sample digestion, 0.1 mL of the blood were measured and put in pre-washed digestion vessels. To the vessels was added 5 mL of nitric acid (atomic absorption spectrometry grade, 30%; Kanto Chemical, Tokyo, Japan), and 1 mL of hydrogen peroxide (Cica reagent, 30%; Kanto Chemical, Tokyo, Japan) in readiness for digestion. The sample digestion was done using a ramped temperature program in a closed microwave system (Speed Wave MWS-2 microwave digestion system; Berghof, Eningen, Germany). The microwave system operating conditions used are given in the Supplementary Materials Table S2. Following cooling, the sample solutions were transferred into 15 mL polypropylene tubes and diluted to a final volume of 10 mL with ultra-distilled and de-ionized water.

The Pb concentration quantification was performed using the ICP-MS (7700 series; Agilent Technologies, Tokyo, Japan), as described by Nakata et al. [28,29] with minor modifications. The operating conditions of ICP-MS were as given in the Supplementary Table S3. The quality control was performed by analysis of DOLT-4 (dogfish liver) (National Research Council of Canada, Ottawa, Canada) certified reference material. Replicate analysis of the reference material gave good recovery rates ranging from 95% to 105%. The detection limit for Pb was 0.001 mg/L.

### 2.4. Laser Ablation Inductively Coupled Plasma Mass Spectrometry (LA-ICP-MS) Analysis of Incisor Teeth of Sprague Dawley and R. rattus Rats

The incisor teeth samples from laboratory and x Pb-exposed wild rats were processed and analyzed according to the method previously described by Ishii et al. [29] with some minor modifications. Briefly, excess tissues and surface contamination of the teeth were cleaned using an ultrasonic bath of L-cysteine for 5 min followed by cleaning in an ultrasonic bath of distilled water and drying at 54 °C for 48 h. Samples were sliced into ~40 µm sections along the longitudinal axis with a diamond blade and polished. The teeth sections were systematically scanned by a focused laser beam with the following parameters—spot diameter: 100 µm, scan speed: 70 µm/sec using LA (NWR213; ESI, Portland, OR, USA)-ICP-QQQ-MS (8800 series; Agilent Technologies) (Agilent Technologies, Inc., Santa Clara, CA, USA). Detailed analytical conditions are presented in Table S4. We reconstructed two-dimensional images from time-resolved analysis data of LA-ICP-MS by iQuant2, as described by Suzuki et al. [32], an in-house developed software. This software shows the localization of elements.

### 2.5. Data Analysis

The data analysis was performed using GraphPad Prism software (Prism 7 for Windows; Version 5.02, GraphPad Software, Inc., CA, USA) and reported as mean and standard deviation (SD). The data were first tested for normality using Kolmogorov–Smirnov test. The data was not normally distributed. We log-transformed the data for statistical analyses and retained the original values in the results and figures for easier interpretation and comparisons with other studies. We applied one analysis of variance (ANOVA) and multiple Tukey’s comparison test as post hoc tests. In the present study, the difference between groups was deemed to be significant at *p* < 0.05 (*) and highly significant at *p* < 0.01 (**). Log transformation of Pb concentrations was also done for the Pearson’s correlations analysis between Pb-B concentration and Pb-T levels. The graphical representations were compiled using GraphPad Prism software.

## 3. Results

### 3.1. Lead in Blood (Pb-B) and Incisors Subdivisions of Laboratory-Exposed Sprague Dawley Rats

**Figure 3:**
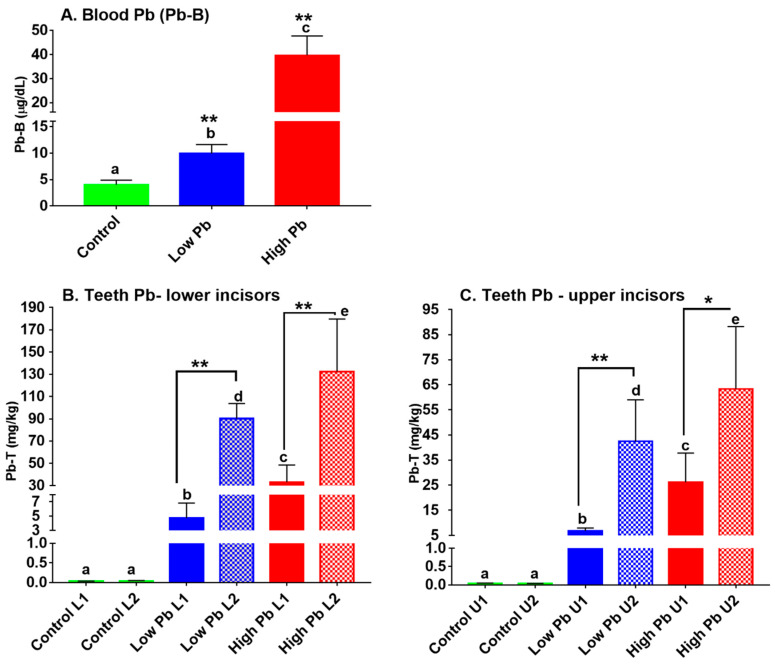
Mean ± SD of Pb-B and Pb-T of laboratory Pb exposed Sprague Dawley rats. (**A**) Pb-B in control (*n* = 6), low Pb (100 mg/L Pb; *n* = 6), and high Pb (1000 mg/L Pb; *n* = 6); (**B**) Pb-T in the lower incisor divisions (L1 and L2) and; (**C**) Pb-T in the upper incisor divisions (U1 and U2). The lower-case letters a, b, c, d, and e represent significant differences among the groups using Tukey’s multiple comparison test (*p* < 0.05). For the Pb-B, ** at *p* < 0.01 represents a significant difference between the control and exposure groups, and for the Pb-T, * at *p* < 0.05 and ** at *p* < 0.01 represent a significant difference between the crown and root of incisor teeth in each group Turkey’s test.

The Pb-B levels increased significantly with the increase in the dose of Pb given through water with the high Pb group having the highest mean concentration of 39.63 ± 8.09 μg/dL, followed by the low Pb group, which had a mean of 9.90 ± 1.71 μg/dL and the control had lowest with 4.01 ± 0.86 μg/dL (Figure 3A). Both the high Pb and low Pb groups accumulated significantly higher Pb levels (*p* < 0.01) when compared to the control (Figure 3A). Further, the Pb-B levels between the low and high groups were also significantly different (*p* < 0.05).

The accumulation of Pb in the teeth subdivisions (Pb-T) in L1 and L2 of lower and U1 and U2 upper incisors are shown in Figure 3B,C, respectively. The accumulation Pb-T in both subdivisions of the lower incisors was in a Pb dose-dependent manner with the high Pb group accumulating higher Pb-T than the low Pb group and the control. The Pb-T in the L2 was significantly higher (Tukey test, *p* < 0.01) than that of L1 for both low Pb and high Pb groups. At low Pb, the Pb-T in L1 was 4.72 ± 2.10 mg/kg, and L2 had 90.17 ± 13.57 mg/kg. At high Pb exposure, L1 had 33.07 ± 15.51 mg/kg, and L2 had 132.40 ± 47.33 mg/kg Pb-T, respectively. Similarly, Pb-T accumulation in the upper incisors and their subdivisions accumulated in Pb dose-dependent manner. The Pb-T in the U1 (6.79 ± 2.03 mg/kg) was significantly lower (*p* < 0.01) than U2 (42.44 ± 16.58 mg/kg) in the low Pb group. Likewise, in the high Pb group, the U1 (26.13 ± 11.63 mg/kg) accumulated lower Pb-T than U2 (63.32 ± 24.89 mg/kg). In addition, we observed that L2 or U2 at lower exposure accumulated significantly higher Pb-T than the L1 or U1 at high exposure (Figure 3B,C).

### 3.2. Relationship between Incisor Teeth Parts Pb and Blood Pb in the Laboratory Exposed Sprague Dawley Rats

**Figure 4:**
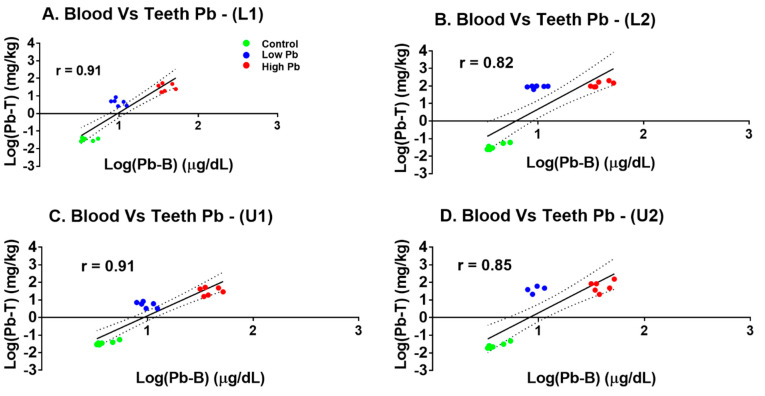
Pearson’s correlations showing the relationship between log-transformed Pb-B and Pb-T crown and root of the lower and upper incisors in the laboratory Pb-exposed Sprague Dawley rats. (**A**) lower incisor (L1) Pb-B versus Pb-T (*r* = 0.91, *p* < 0.01); (**B**) lower incisor (L2) Pb-B versus Pb-T (*r* = 0.82, *p* < 0.01); (**C**) upper incisor (U1) Pb-B versus Pb-T (*r* = 0.91, *p* < 0.01) and (**D**) upper incisor (U2) Pb-B Vs Pb-T (*r* = 0.85, *p* < 0.01).

Figure 4 shows the relationship between the Pb-T in the lower and upper incisors root and crown subdivisions with the Pb-B concentration in the laboratory Sprague Dawley rats. In the present study, positive log-transformed Pearson’s correlations between the Pb-B and Pb-T across all the exposed groups in the root and crown of the lower and upper incisors were observed. Much stronger correlations between Pb-T and Pb-B were recorded in the root of both lower and upper incisors than in the crown. The correlations were significant, namely, lower incisor (L1) Pb-T versus Pb-B (*r* = 0.91, *p* < 0.01), lower incisor (L2) Pb-T versus Pb-B (*r* = 0.82, *p* < 0.01), upper incisor (U1) Pb-T versus Pb-B (*r* = 0.91, *p* < 0.01), and upper incisor (U2) Pb-T versus Pb-B (*r* = 0.85, *p* < 0.01), as shown in Figure 4A–D.

### 3.3. Lead in Blood and Teeth and Their Relationship in the Lead-Exposed R. rattus Rats

**Figure 5:**
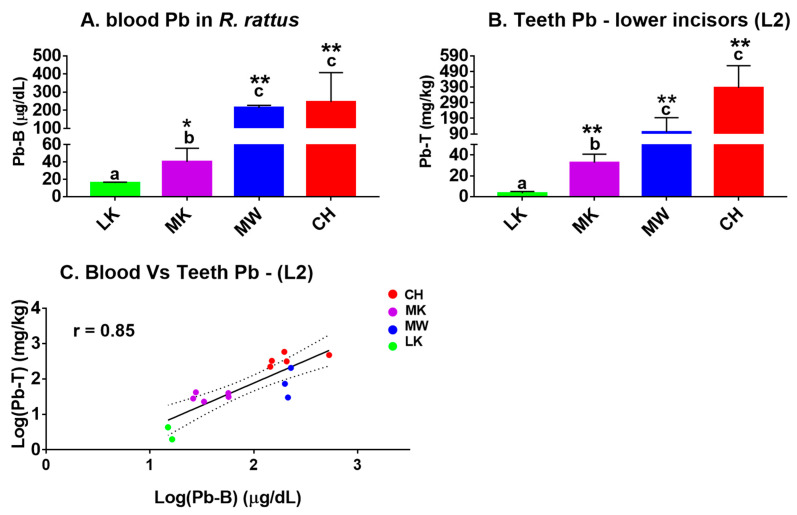
Mean ± SD of Pb-B and Pb-T of Pb-exposed *R. rattus* rats. (**A**) Pb-B from rats sampled from Lukanga (LK; *n* = 2); Makululu (MK; *n* = 5); Mutwe Wansofu (MW; *n* = 3) and Chowa (CH; *n* = 5). (**B**) Pb-T from rats sampled from LK (*n* = 2); MK (*n* = 5); MW (*n* = 3), and CH (*n* = 5); The lower case letters a, b, and c represent significant differences among the groups using the Tukey’s multiple comparison test (*p* < 0.05). For the both Pb-B and the Pb-T, * at *p* < 0.05 and ** at *p* < 0.01 represent significant difference between the LK site (control group) and other groups sampled closer to the former Pb–Zn mine using the Turkey’s test. (**C**) Log-transformed Pearson’s correlations of Pb-B versus Pb-T in *R. rattus* rats (*r* = 0.85, *p* < 0.01).

Figure 5A shows the Pb-B in wild rats sampled from different sites within varying distances from the closed Pb–Zn mine as a reference point as shown in Figure 1. The accumulation of Pb-B differed significantly among the sites with the highest Pb-B levels seen in rat samples captured closer to the old mine and lowest in rat samples collected furthest from the mine (Figure 5A). Quantitatively, the CH site group had a mean Pb-B of 245.40 ± 161.90 μg/dL, the MW site group had 213.20 ± 14.22 μg/dL, the MK site group had 40.11 ± 15.56 μg/dL and the LK site group had 15.60 ± 0.99 μg/dL. Furthermore, in reference to the control site sample (LK), the Pb-B levels in known contaminated sites were significantly higher in MK (*p* < 0.05), MW (*p* < 0.01), and CH (*p* < 0.01) groups. No difference in Pb-B levels in the CH and MW groups was observed (Figure 5A).

Figure 5B shows the accumulation pattern of Pb-T in the crown part of the lower incisor (L2) of *R. rattus* rats exposed to Pb in their natural environment. Because an application of our laboratory results showed a higher Pb level in the L2 section compared to the L1 section of incisors (Figure 3B,C), only L2 Pb levels in *R. rattus* rats are shown. The accumulation Pb-T was higher in the rats that were captured closer to the closed Pb–Zn mine had higher Pb-T levels than those captured further away from the closed Pb–Zn mine. Quantitatively, the CH group had the highest levels with Pb-T of 383.60 ± 144.10 mg/kg, followed by MW group with Pb-T of 102.60 ± 91.60 mg/kg, MK with 32.66 ± 8.02 mg/kg, and the least Pb-T was in the LK group with 3.17 ± 1.69 mg/kg. The Pb-T concentrations among the groups were statistically significant (*p* < 0.05), as shown by lower case letters (Figure 5B). In addition, the exposed groups were significantly different from the assigned control (LK) group (*p* < 0.01). In the present study, the Pearson’s correlation analysis between Pb-B and Pb-T of log-transformed data in the crown of lower incisors in wild rats exposed to Pb was positive (*r* = 0.85, *p* < 0.01), as shown (Figure 5C).

### 3.4. Lead Distribution in the Incisor Teeth of the Lead-Exposed Laboratory Sprague Dawley and Wild R. rattus Rats Using LA-ICP-MS

**Figure 6:**
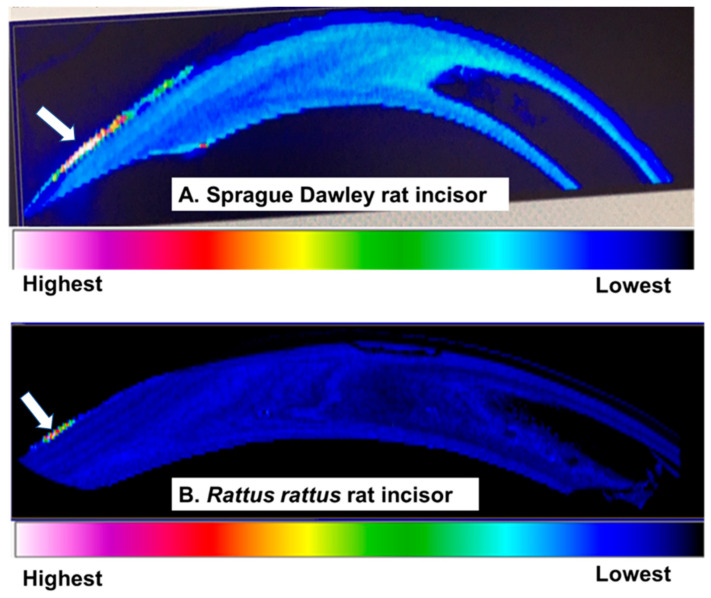
Pb distribution in the incisor teeth of the lead-exposed laboratory Sprague Dawley and wild exposed (*R. rattus)* rats using LA-ICP-MS. Homogenous distribution of Pb in the pulp and dentine of the with inhomogeneous distribution of Pb-T characterized by intense deposits of Pb in the exterior enamel (arrow). (**A**) Sprague Dawley and (**B**) *R. rattus* rats in incisors.

The local distribution of the Pb mappings using LA-ICP-MS in the incisor teeth of both laboratory and wild rats exposed to Pb is shown in Figure 6A,B, respectively. A homogenous distribution of Pb was observed in the greater portion of the tooth from the root extending to the pulp and the dentine in both laboratory and wild rodent teeth samples (Figure 6A,B). On the other hand, an inhomogeneous distribution of Pb was found in the surface enamel near the tip of the incisor crown having an intense localized distribution of Pb. The distribution of Pb in both laboratory and wild rodent teeth was similar (Figure 6A,B).

## 4. Discussion

The present study focused on Pb quantities in the rodent incisor tooth as a biomarker of Pb and its applicability in Pb exposure screening. The most striking observation was the high accumulation of Pb-T in the crown of the upper and lower incisor teeth than the roots across all levels of exposure in laboratory Pb-exposed Sprague Dawley rats. The inherent incisor tooth structural variations between the root and crown due to the larger part of the dental root pulp and higher dentine crown mass, as well as dentine-enamel ratio, could be the key factors in the accumulation of Pb-T differences [33]. The rodent incisors are predominantly dentine with a thin layer of enamel located only in the front part of the tooth [30]. The influence of the presence of the large dentine mass in the crown of incisor teeth may have been one of the contributing factors behind the L2 or U2 in the low Pb group accumulating much Pb than the L1 or U1 in the high Pb exposure group. The current findings seem to agree with other studies that demonstrated that Pb is highly accumulated in the dentine part of the teeth [14,34,35,36].

The Pb-T in both the root and the crown of the lower incisors and upper incisor teeth and the Pb-B accumulation in the Sprague Dawley rats following laboratory exposure were in a Pb dose-dependent pattern. These findings suggest that rodent incisors could be a useful indicator of exposure to Pb, as was reported in goats [34] and rats [16]. Furthermore, strong positive correlations between Pb-B and Pb-T were found in the Pb-exposed laboratory rats, similar to what has been reported in humans [33,37]. Taken together, our results in the laboratory Pb-exposed Sprague Dawley rats showed that Pb-T in the crown incisor analysis may provide some advantages in assessing Pb exposure.

In the *R. rattus* rat species that were used as sentinel animals around the closed Pb–Zn mine in Kabwe, Zambia, the accumulation of Pb-T in L2 and Pb-B were linked to the distance in reference to the point source. Moreover, there was a strong positive correlation between Pb-T in L2 and Pb-B in the wild of rats, suggesting that the incisor crown would be a good predictor of Pb exposure just like Pb in blood. This was in agreement with the pattern of Pb-T accumulation reported in deciduous teeth of children living near a lead-acid battery smelter [38]. Comparatively, our results were also in tandem with surface enamel Pb-T in children, where much Pb-T accumulation was reported in polluted areas than in less polluted areas [39]. Furthermore, the Pb-T in the crown of the sentinel rats in the present study corroborated the findings of Pb concentrations in soils [25], and Pb-B in free-roaming dogs [40] and free-range chickens [41] sampled within the vicinity of the former Pb–Zn mine. Taken together, the current findings indicate that the rodent tooth incisor crown may be a useful tool for environmental Pb exposure monitoring.

The Pb-T in the crown incisors were relatively higher than the Pb-B, corroborating reports that indicated that Pb-T were better indicator exposure and cumulative Pb body burden than Pb-B [42]. The high Pb-B levels observed in wild rats were however not surprising because they were recorded from the Chowa and Mutwe Wansofu sampling sites that were near to the mine where children with very high Pb-B were previously reported [4]. On the other hand, we observed that the lower the Pb-B level, the lower the Pb-T, which adds further merits to the use of L2 sample as a biomarker of exposure. While the duration of exposure in wild rats *(R. rattus)* may not be clearly known, findings in the laboratory Pb exposed rats that were exposed for eight weeks suggest that the teeth may be useful both for chronic Pb exposure and in sub-chronic conditions exposure where blood levels are elevated.

The Pb-T distribution mapping using LA-ICP-MS in the lower incisors of the Pb-exposed laboratory and wild rats was performed for the first time. Interestingly, the distribution of Pb-T was homogeneously distributed in the dental pulp, dentine, and the greater part of enamel except on the front tip part of the incisor crown with enamel in both laboratory and wild rats. The intense Pb localization seen on the tip of the incisor crown with an enamel surface was in contrast to other studies that demonstrated that calcified tissue layers in direct contact or in proximity to vascular tissues accumulated much more Pb than those further away in bones [29] and teeth around the circumpulpal dentine [18,34,43]. However, the present findings agreed in part with a report in human incisor teeth samples where most of the Pb were primarily deposited in the secondary dentine region close to the pulp, and secondarily, at surface enamel [12]. Moreover, the observed high intensities of Pb in the outer part of incisors were only on the front side, the only side bearing enamel in rodent incisors [30]. This phenomenon has been demonstrated in both erupted and non-erupted teeth that highly accumulated Pb in the outer enamel surface and with a gradual reduction of Pb in the deeper layers of the enamel [19,39,44,45]. Taken together, the characteristic enamel surface Pb accumulation in the crown incisors further supports the use of the crown incisor as an alternative biomarker of Pb exposure screening in wild and laboratory rats, with the former having merits for use as a sentinel marker of exposure.

The current study limitation bordered around wild rats sampling. The small number of samples for the LK (*n* = 2) and MW (*n* = 3) after the exclusion of other species after genomic sequencing except the *R. rattus* species is the notable limitation. The logistics and travel restrictions could not permit re-sampling to increase the sample size of the *R. rattus*. Further validation of the rat incisor crown as a suitable alternative biomarker to blood-Pb for environmental Pb exposure assessment with a much larger sample size is recommended. Notwithstanding, the current study has revealed that the crown subdivision of the incisor rodents could be a suitable biomarker of Pb exposure. The use of the incisor crown subdivision has merits because it is easy to extract and is stable for preservation purposes [10]. Besides, the inherent advantage of teeth over the blood sample matrix in its ability to retain Pb after a month or more after the source is removed makes it an attractive alternative biomarker of Pb exposure. In the current study, wild rodents were trapped from residential areas, and sometimes in houses, making them suitable sentinel markers for human exposure, especially children with hand-to-mouth activities. Furthermore, targeting the crown incisor for Pb exposure assessment without targeting the whole incisor tooth will maximize time, resources, and increase the chances of detection of Pb in sentinel wild rats.

## 5. Conclusions

The crown and the root of both lower and upper incisor teeth Pb-T in laboratory Pb- exposed Sprague Dawley rats accumulated Pb in a dose-dependent manner with the crown accumulating much more Pb-T than the root, suggesting that the crown may be a superior marker of Pb exposure than the root. Furthermore, the Pb-T accumulation in the crown of the lower incisors (L2) of wild rats discriminated the varying distances of sampling sites in relation to the Pb–Zn mine point source in Pb exposed *R. rattus* rats. In addition, the strong positive correlations between Pb-B and Pb-T observed in both laboratory and wild rats exposed to Pb support the possibility of rodent teeth as a useful tool for environmental assessment of Pb exposure. The high Pb-T in the crown compared to the root and the highly localized distribution of Pb in the incisor crown bearing enamel, as observed from the LA-ICP-MS mapping, indicate that the crown subdivision of the incisor tooth may be adequate for its use in sentinel rodents for Pb exposure assessment. Further studies are required to validate the rat incisor crown as a suitable alternative biomarker to blood-Pb for environmental Pb exposure assessment.

## Data Availability

The datasets generated and analyzed during the current study are are available from the corresponding author on reasonable request.

## Supplementary Materials

The following are available online at https://www.mdpi.com/1660-4601/18/2/767/s1, Table S1: Wild rat species, Table S2: Microwave operating conditions for teeth and blood digestion, Table S3: Detailed analytical conditions of ICP-MS, Table S4: Detailed analytical conditions of LA-ICP-MS.

## Appendix A. Wild Rat Species Identification

The species of the wild rats sampled were identified using genomic DNA sequencing. Genomic DNA was extracted from liver samples of wild rodents with Wizard^®^ Genomic DNA Purification Kit (Promega Corporation Madison, WI, USA). Species discrimination was done following a slight modification of the method described by Robins et al. [24]. To amplify Cytochrome b, 762 bp (*Cyt-b)* gene with accession number: JX887164.1, the primers used were Forward primer (5′-GGTGAAGGCTTCAACGCCAACCCTA-3′) and Reverse primer (5′-TAGAATATCAGCTTTGGGTGTTGATGG-3′). The polymerase chain reaction (PCR) thermal regime for all amplifications was one initial denaturation step of 94 °C for 2 min and 35 cycles of denaturation at 94 °C for 30 min, annealing at 60 °C for 30 min and extension at 72 °C for 1 min; and a final extension step of 72 °C for 6 min with a thermal cycler (iCycler, Bio-Rad, Hercules, CA, USA) and EmeraldAmp MAX PCR Master Mix (Takara Bio Inc., Shiga, Japan). The volume of PCR mix was 10 µL containing in 50 ng genomic DNA, 5 µL of EmeraldAmp MAX PCR Master Mix and 0.2 µM of each forward and reverse primer. The amplified products were purified by QIAquick^®^ PCR Purification kit (Qiagen, Hilden, Germany). The sequence reaction was done by Fasmac Co. Ltd. (Kanagawa, Japan). From these sequences done by Fasmac Co. Ltd., the rat species were identified through blast analysis using the National Center for Biotechology Information (NCBI) website. Based on the results 15 *Rattus rattus* rats in total were identified and used for the study. And from each sampling site, the following numbers of paired blood and lower incisors teeth of *R. rattus* rats in brackets were included in the study, namely, Lukanga (LK) (*n* = 2); Mutwe Wansofu (MW) (*n* = 3); Chowa (CH) (*n* = 5); and Makululu (MK) (*n* = 5), respectively (Table S1).
