# Supplementary material for: An Investigation of the Wild Rat Crown Incisor as an Indicator of Lead (Pb) Exposure Using Inductively Couple Plasma Mass Spectrometry (ICP-MS) and Laser Ablation ICP-MS
Source: Int J Environ Res Public Health. 2021 Jan 18;18(2):767. doi: 10.3390/ijerph18020767 (PMC7830958; doi:10.3390/ijerph18020767)
Supplement: Supplementary file 1 [file ijerph-18-00767-s001.pdf]

Table S1. Wild rat species

Table S2. Microwave operating conditions for teeth and blood digestion

Table S3. Detailed analytical conditions of ICP-MS.

Table S4. Detailed analytical conditions of LA-ICP-MS.

Table S1. Wild rat species

No.	ID	Species	Sex	Site
1	LK1	*Rattus rattus*	Male	Lukanga
2	LK2	*Rattus rattus*	Female	Lukanga
3	CH1	*Rattus rattus*	Female	Chowa
4	CH2	*Rattus rattus*	Female	Chowa
5	CH3	*Rattus rattus*	Male	Chowa
6	CH4	*Rattus rattus*	Male	Chowa
7	CH5	*Rattus rattus*	Male	Chowa
8	MK1	*Rattus rattus*	Female	Makululu
9	MK2	*Rattus rattus*	Male	Makululu
10	MK3	*Rattus rattus*	Female	Makululu
11	MK4	*Rattus rattus*	Male	Makululu
12	MK5	*Rattus rattus*	Male	Makululu
13	MW1	*Rattus rattus*	Female	Mutwe Wansofu
14	MW2	*Rattus rattus*	Male	Mutwe Wansofu
15	MW3	*Rattus rattus*	Male	Mutwe Wansofu

Samples analyzed after exclusion of the non- *R. rattus* rat species, final sample size was n = 2 (LK), n = 5 (CH), n = 5 (LK), n = 5 (MK) and n = 3 (MW), respectively.

Table S2. Microwave operating conditions for teeth and blood digestion.

Teeth		Blood	
Temperature (ºC)	Time (min)	Temperature (ºC)	Time (min)
160	5	160	5
190	20	190	10
200	20	75	10
100	5		

Table S3. Detailed analytical conditions of ICP-MS.

Parameter	Value
RF Power	1500 W
Argon gas pressure	600 kPa
Cell gas (Helium)	100 kPa
Peak pattern	1
Replicates	3
Sweeps/replicate	100
Stabilization time	30 s

Table S4. Detailed analytical conditions of LA-ICP-MS.

LA system (NWR213, ESI, Portland, OR, USA)	
Wavelength, nm	213
Pulse duration, ns	4
Fluence	2.7 J/cm^2
Repetition rate	10 Hz
Spot diameter	100 μm (zoomed version: 20 μm)
Scan speed	500 μm/ sec (zoomed version: 20 μm/sec)
Ablation mode	line scan
Carrier He gas flow rate	0.8 L/min
Make up Ar gas flow rate	0.8 L/min
ICP-QQQ-MS (8800 series, Agilent Technologies, Tokyo, Japan)	
RF power	1550 W
Plasma Ar gas flow rate	15 L/min
Auxiliary Ar gas	not used
Collision	not used
MS/MS	not used
Integration time	0.01 sec for ^{206}Pb, ^{207}Pb, ^{208}Pb, and 0.005 sec for other isotopes
Measured Isotopes	^{13}C, ^{25}Mg, ^{31}P, ^{43}Ca, ^{55}Mn, ^{57}Fe, ^{65}Cu, ^{66}Zn, ^{206}Pb, ^{207}Pb, ^{208}Pb
